# Ethnic differences of small turtle ownership in the United States: a descriptive analysis of Porter Novelli Estilos and SpringStyles surveys, 2021 and 2023

**DOI:** 10.3389/fpubh.2026.1920888

**Published:** 2026-10-15

**Authors:** Marisa Wong, G. Sean Stapleton, Julio Dicent Taillepierre, Katharine Benedict

**Affiliations:** 1 Division of Foodborne, Waterborne, and Environmental Diseases, Centers for Disease Control and Prevention, Atlanta, GA, United States; 2 Oak Ridge Institute for Science and Education, Oak Ridge, TN, United States; 3 Office of Health Equity, Centers for Disease Control and Prevention, Atlanta, GA, United States

**Keywords:** health disparities, outbreak, prevention communication, Salmonella, zoonotic

## Abstract

**Introduction:**

Turtles with shell length <4 inches (“small turtles”) are prohibited for sale as pets in the United States because of their potential to expose people, particularly children, to *Salmonella*. In previous multistate turtle-associated salmonellosis outbreaks, people of Hispanic or Latino ethnicity have been disproportionately affected. This study aimed to describe the demographic characteristics of United States adults who own small turtles and to document the purchase locations of these small turtles.

**Methods:**

We analyzed data from a nationally representative survey of Hispanic United States adults and a nationally representative survey of all United States adults. Both surveys included similar questions about small turtle ownership and purchase locations. We compared frequencies of responses from these two surveys using weighted cross-tabulations.

**Results:**

Twenty percent (95% CI: 16.1–24.1%) of Hispanic or Latino people reported ever owning small turtles, compared with 14% (95% CI: 12.8–14.9%) of respondents of all other racial and ethnic groups in the United States. Sixty four percent (95% CI: 54.3–74.4%) of Hispanic respondents reported purchasing their small turtles from pet stores.

**Conclusion:**

A greater percentage of Hispanic or Latino adults have ever owned small turtles compared to other United States adults, which may contribute to the overrepresentation of Hispanic and Latino people in turtle-associated outbreaks. Additional research could help elucidate factors contributing to higher turtle ownership among Hispanic and Latino people and how knowledge, attitudes, and practices of small turtle owners affects salmonellosis risk. Such information could aid in focusing public health prevention efforts on populations who reported higher weighted prevalence of owning small turtles.

## Introduction

Turtle-associated salmonellosis is a public health problem in the United States that particularly affects children and was documented since the 1960s (1). To reduce occurrence of these illnesses, the United States Food and Drug Administration implemented a federal ban on the sale and distribution of turtles with shell length of <4 inches (hereafter, “small turtles”) as pets (2). Despite this ban and some state prohibitions, salmonellosis outbreaks linked to contact with small turtles continue to occur yearly (3–5). These outbreaks have disproportionately affected people of Hispanic ethnicity. In eight multistate outbreaks linked to small turtles from 2011–2013, 45% of patients (85 of 191 patients included in all outbreaks) were Hispanic (ranging between 0% [0 of 7 patients] and 71% [34 of 48 patients]) (6). In 2015, nearly 70% of patients included in salmonellosis outbreaks linked to small turtles were Hispanic (4). More recent outbreaks have similarly affected Hispanic people (7). In comparison, the proportion of people in the United States identifying as Hispanic or Latino was estimated to range between 18.8–19.4% during 2021–2023 (8).

To better understand the populations at risk of small turtle-associated salmonellosis and to inform strategies to prevent these illnesses, we used two nationally representative Porter Novelli surveys to describe small turtle ownership and small turtle purchase locations across racial and ethnic groups.

## Materials and methods

Estilos is a Porter Novelli online survey of United States adults of Hispanic ethnicity, administered by Offerwise’s QueOpinas panel and recruited via English and Spanish network television. Offerwise QueOpinas released the survey to 2,669 people ≥18 years and collected results during the predetermined time period of October 1, 2021–November 9, 2021. Respondents were not required to participate in the survey. Offerwise QueOpinas provided a reward worth $15 to respondents who completed the survey. Survey data were weighted based on United States Census American Community Survey proportions according to sex, age, household income, household size, education, census region, country of origin, and the respondent’s acculturation. Acculturation was defined by survey administrators based on the respondent’s cultural self-identification, years lived in the United States, Spanish language media use, and language spoken at home (9). Estilos’ acculturation model assigns point values based on respondent’s answer choices to these survey questions (Supplementary Table S1).

SpringStyles is a Porter Novelli ConsumerStyles survey of United States adults, administered by Ipsos’ KnowledgePanel and recruited by mail. KnowledgePanel released the survey to 11,390 people and collected responses during March 23, 2023–April 14, 2023. KnowledgePanel offered a reward worth $5 to respondents who completed the survey. Survey data were weighted to resemble the United States Current Population Survey proportions on sex by age, household income, race and ethnicity, household size, education, census region, metro status, and parental status (10).

We developed questions for both surveys with the primary purpose of obtaining information that could inform our understanding of small turtle-associated salmonellosis. Both surveys included similar questions about demographic characteristics and small turtle ownership and purchase (Supplementary Table S1). We descriptively analyzed whether respondents currently, previously, or never owned a small turtle using SAS 9.4 Unicode Support (SAS Institute, Inc., Cary, NC) survey frequency procedures, to obtain weighted proportions and 95% confidence intervals (CI) of respondents who selected each answer choice. We compared weighted proportions based on assessment of 95% CIs. Proportions and confidence intervals were generated separately for each survey, and we advise caution when interpreting confidence intervals between separate surveys. No additional statistical tests were performed to compare the results of the two surveys in order to avoid conflating differences between the two surveys and to provide the most cautious descriptive analysis possible. We combined responses of current small turtle ownership with previous small turtle ownership into the category of “ever owning” a small turtle to increase weighted estimate stability. Similarly, we combined small turtle purchase locations into four categories: pet store, transient vendor (presenting purchase at a roadside-vendor or flea market), non-commercial acquisition (including catching the turtle in the wild, adoption from an animal shelter or rescue, or receiving the turtle from a friend or family member), and unknown. We labeled SpringStyles respondents as “Multiple races” if respondents selected “I identify with more than one race equally” when asked “What race or ethnicity do you most identify with?” We cross-tabulated small turtle ownership with demographic variables, and we obtained the frequency of small turtle procurement locations. For the Estilos survey only, we calculated the frequency of respondent answers to cultural self-identification, years lived in the United States, Spanish language media use, and language spoken at home; then, we stratified these responses by small turtle ownership status. Data visualizations were produced using R Statistical Software version 4.3.1 (11).

No identifying information was included in the analyzed data sets. As a result, analyses of data from the 2021 Estilos and 2023 SpringStyles surveys were declared exempt by CDC’s institutional review board. This activity was reviewed by the United States Centers for Disease Control and Prevention and was conducted in a manner consistent with applicable federal law and CDC policy, including 45 C. F. R. part 46; 21 C. F. R. part 56; 42 U. S. C. Sect. 241(d); 5 U. S. C. Sect. 552a; 44 U. S. C. Sect. 3,501 et seq.

## Results

In 2021, 1,000 (37%) individuals completed the Estilos survey of 2,669 who were requested to complete it (Table 1). In 2023, 6,694 (59%) individuals completed the SpringStyles survey of 11,390 who were requested to complete it. Among Estilos respondents (which only included Hispanic people), 20.1% (95% CI: 16.1–24.1%) reported ever owning a small turtle (Figure 1). Among SpringStyles respondents reporting Hispanic ethnicity, 20.8% (17.0–24.6%) reported ever owning a small turtle. Among SpringStyles respondents not reporting Hispanic ethnicity, 13.8% (95% CI: 12.8–14.9%) reported ever owning a small turtle. Among SpringStyles respondents including those reporting Hispanic ethnicity, 14.7% (13.7–15.7%) reported ever owning a small turtle. Among SpringStyles respondents who identify with multiple races, 23.7% (16.4–31.0%) reported ever owning a small turtle.

**Table 1:** Demographic information of Estilos and SpringStyles respondents, stratified by survey and small turtle ownership status, United States, 2021 and 2023^†,‡^.

Demographic information	Estilos (*N* = 1,000)		SpringStyles (*N* = 6,694)^§^	
	Ever owned a small turtle	Never owned a small turtle	Ever owned a small turtle	Never owned a small turtle
	% of *n* (95% CI)	% of *n* (95% CI)	% of *n* (95% CI)	% of *n* (95% CI)
All respondents	20.1% (16.1–24.1%)	79.9% (75.9–83.9%)	14.7% (13.7–15.7%)	85.3% (84.3–86.3%)
Race and ethnicity				
Hispanic or Latino	20.1% (16.1–24.1%)	79.9% (75.9–83.9%)	20.8% (17.0–24.6%)	79.2% (75.4–83.0%)
American Indian or Alaska Native	—	—	13.2% (2.9–23.6%)^¶^	86.8% (76.4 – 97.1%)
Asian	—	—	10.5% (6.3–14.7%)	89.5% (85.3–93.7%)
Black or African American	—	—	12.7% (9.6–15.8%)	87.3% (84.2–90.4%)
Native Hawaiian or Pacific Islander	—	—	8.8% (0–23.0%)^¶^	91.2% (77.0 – 100%)^¶^
White	—	—	14.0% (12.8–15.1%)	86.0% (84.9–87.2%)
Multiple races	—	—	23.7% (16.4–31.0%)	76.3% (69.0–83.6%)
Age group (years)				
18–24	25.9% (13.7–38.2%)	74.1% (61.8–86.3%)	10.9% (7.2–14.5%)	89.1% (85.5–92.8%)
25–34	24.6% (15.5–33.8%)	75.4% (66.2–84.5%)	12.7% (10.1–15.3%)	87.3% (84.7–89.9%)
35–44	24.6% (16.4–32.8%)	75.4% (67.2–83.6%)	11.7% (9.6–13.8%)	88.3% (86.2–90.4%)
45–54	18.7% (10.2–27.2%)	81.3% (72.8–89.8%)	13.8% (11.5–16.1%)	86.2% (83.9–88.5%)
55–64	14.1% (5.8–22.4%)^¶^	85.9% (77.6 – 94.2%)	16.6% (14.2–19.1%)	83.4% (80.9–85.8%)
65 or older	2.3% (0–6.1%)^¶^	97.7% (93.9 – 100%)	19.9% (17.7–22.0%)	80.1% (78.0–82.3%)
Education level				
Less than High School	24.1% (14.8–33.5%)	75.9% (66.4–85.2%)	17.6% (13.4–21.9%)	82.4% (78.1–86.6%)
High School	16.6% (9.6–23.7%)	83.4% (76.3–90.4%)	15.5% (13.5–17.6%)	84.5% (82.4–86.5%)
Some College	16.4% (9.3–23.6%)	83.6% (76.4–90.7%)	16.5% (14.5–18.4%)	83.5% (81.6–85.5%)
College Degree or higher	23.9% (17.4–30.5%)	76.1% (69.5–82.6%)	11.9% (10.6–13.3%)	88.1% (86.7–89.4%)
Parental status				
Parent to any child(ren) < 5 years	21.1% (13.8–28.4%)	78.9% (71.6–86.2%)	13.5% (10.1–17.0%)	86.5% (83.0–89.9%)
Parent to only child(ren) 5–17 years	30.8% (22.4–39.2%)	69.2% (60.8–77.6%)	13.3% (11.5–15.2%)	86.7% (84.8–88.5%)
Parent to any children at the time of survey	27.0% (21.0–33.0%)	73.0% (67.0–79.0%)	13.4% (11.7–15.1%)	86.6% (84.9–88.3%)
Not a parent	15.2% (10.1–20.4%)	84.8% (79.6–89.9%)	15.2% (14.0–16.4%)	84.8% (83.6–86.0%)

^†^Results from Estilos and SpringStyles surveys are nationally representative and weighted based on the United States Census American Community Survey and the United States Current Population Survey, respectively.

^‡^Percentages and confidence intervals are rounded to the nearest tenths place. Percentages represent the proportion of respondents that selected the specific demographic and turtle-based question answer choice.

^§^35 Springstyles respondents (0.5%; 0.3–0.7%) refused to answer the small turtle ownership question and were not shown in this table.

^¶^Relative standard error was greater than 30% of the weighted estimate; therefore, these weighted estimates and 95% CI’s should be interpreted with caution (8, 12).

**Figure 1:**
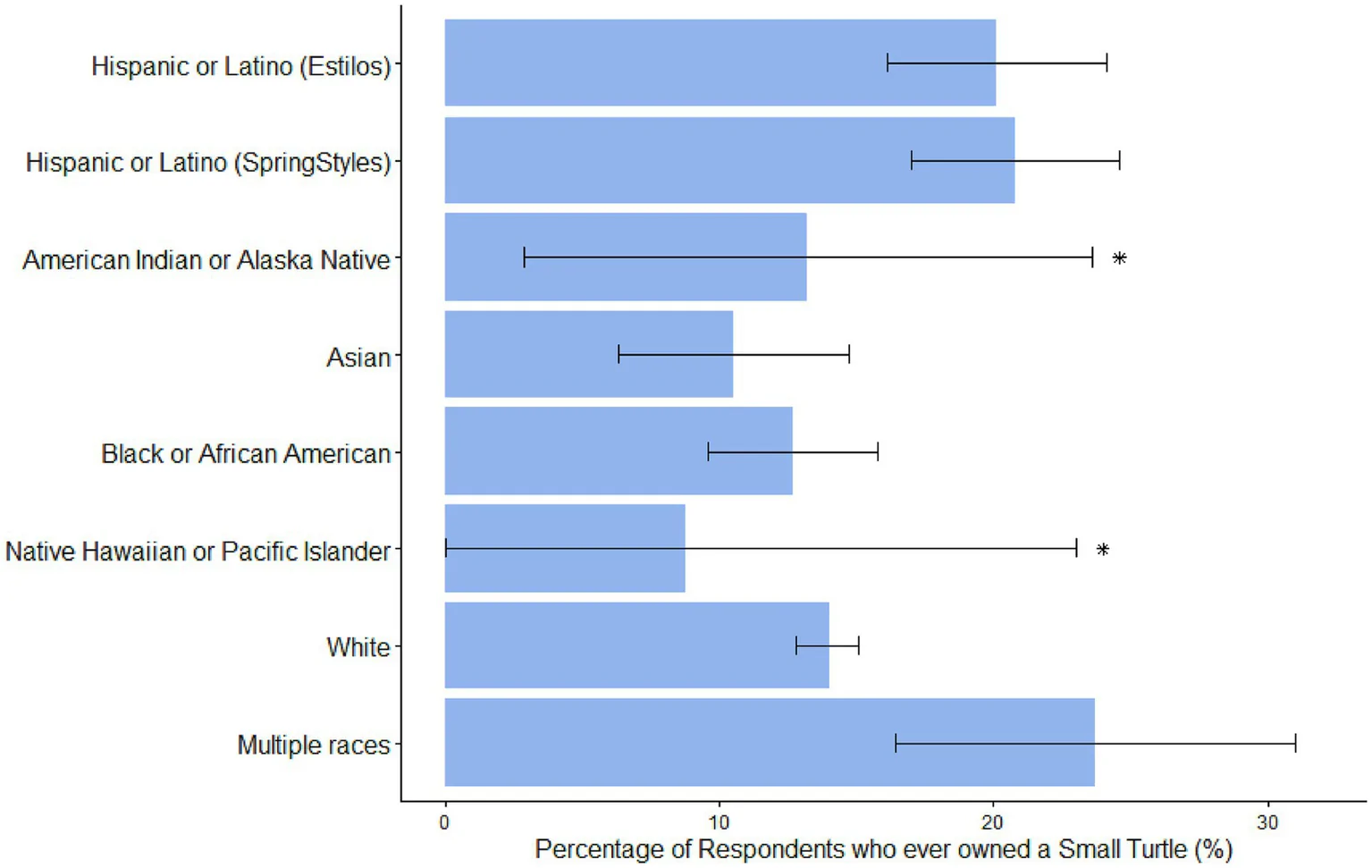
Small turtle ownership by race and ethnicity – United States, 2021 and 2023. In 2021, 1,000 individuals completed the Estilos survey. In 2023, 6,694 individuals completed the SpringStyles survey, 6,659 of whom provided race and ethnicity information. Percentages represent the proportion of respondents in their respective racial or ethnic group that ever owned (currently or previously) a small turtle. Error bars represent the 95% confidence intervals calculated for the weighted percentages. Hispanic or Latino respondents from both surveys are included, and the specific survey is noted in parentheses. Data for other racial and ethnic groups were only captured in the SpringStyles survey. Asterisks represent weighted estimates with >30% relative standard error; therefore, these weighted estimates and 95% CI’s should be interpreted with caution (8, 12).

Hispanic respondents in the Estilos survey that were 25–34 or 35–44 years of age reported higher weighted prevalence of ever owning a small turtle compared with respondents of all races and ethnicities in the SpringStyles survey in those age groups (Table 1). Similarly, Hispanic respondents in the Estilos survey that were parents to any children under age 17 were reported higher weighted prevalence of ever owning a small turtle (27.0%; 95% CI: 21.0–33.0%) compared with respondents of all races and ethnicities that were parents to any children under age 17 in the SpringStyles survey (13.4%; 95% CI: 11.7–15.1%). There were no differences between the two surveys regarding small turtle ownership and education level (Table 1).

Estilos respondents provided responses for their cultural self-identification, years lived in the United States, media habits, and language spoken at home (Table 2). For all Estilos respondents, the greatest proportion reported identifying “somewhat/much closer” to Hispanic or Latino culture than US culture, living in the United States for 20 years or more, consuming Spanish and English media equally, or speaking Spanish mostly or only at home. Among Estilos respondents who ever owned a small turtle, respondents most frequently identified as “somewhat/much closer” to Hispanic or Latino culture than US culture, lived in the United States for 0–19 years, used “somewhat/mostly” Spanish media, or spoke Spanish mostly or only at home.

**Table 2:** Hispanic culture characteristics among Estilos respondents that contribute to Estilos’ acculturation model^†,‡^.

Hispanic culture characteristics	All Respondents (*n* = 1,000) % of all respondents (95% CI)	Respondents who ever owned a small turtle (*n* = 201) % of *n* (95% CI)	Respondents who never owned a small turtle (*n* = 799) % of *n* (95% CI)
Cultural Identification			
Somewhat/much closer to Hispanic/Latino Culture	55.5% (49.7–61.2%)	67.1% (57.6–76.6%)	52.5% (46.0–59.1%)
Equally close to both cultures	35.6% (29.8–41.5%)	25.8% (17.4–34.2%)	38.1% (31.3–44.9%)
Somewhat/much closer to US Culture	8.9% (5.7–12.1%)	7.1% (1.6–12.6%)^§^	9.4% (5.6–13.1%)
Years Lived in the United States			
0–19	44.2% (38.8–49.5%)	58.5% (47.9–69.1%)	40.6% (34.7–46.4%)
≥20	55.8% (50.5–61.2%)	41.5% (30.9–52.1%)	59.4% (53.6–65.3%)
Media Habits			
Somewhat/mostly Spanish media	36.0% (31.0–41.0%)	47.0% (36.2–57.8%)	33.2% (27.8–38.7%)
Spanish and English media equally	39.4% (34.2–44.6%)	37.3% (27.1–47.6%)	39.9% (33.9–46.0%)
Somewhat/mostly English media	24.6% (18.6–30.5%)	15.6% (7.5–23.8%)	26.8% (19.8–33.9%)
Language Spoken at Home			
Spanish mostly/only	44.7% (39.3–50.1%)	47.5% (36.8–58.2%)	44.0% (37.8–50.1%)
Spanish and English equally	35.0% (29.9–40.1%)	41.4% (30.9–52.0%)	33.3% (27.6–39.1%)
English mostly/only	20.4% (14.4–26.3%)	11.1% (3.3–18.9%)^§^	22.7% (15.7–29.7%)

^†^Estilos’ Acculturation Model assigns point values to answer choices regarding a respondent’s cultural identification, years living in the United States, media habits, and language spoken at home.

^‡^Percentages and confidence intervals are rounded to the nearest tenth place. Percentages represent the proportion of respondents that selected the specific acculturation variable and turtle-based question answer choice.

^§^Relative standard error was greater than 30% of the weighted estimate; therefore, these weighted estimates and 95% CI’s should be interpreted with caution (8, 12).

Pet stores were the location most commonly reported for purchasing small turtles (Table 3). A majority of Estilos respondents obtained their small turtle from a pet store (64.3%; 95% CI: 54.3–74.4%), and the largest proportion of Hispanic SpringStyles respondents also obtained their turtle from a pet store (36.4%; 95% CI: 29.0–43.9%). Other notable sources of small turtles among Estilos respondents include non-commercial acquisition (21.4%; 95% CI: 14.2–28.6%) and transient vendors (19.5%; 95% CI: 10.8–28.1%) such as roadside vendors and flea markets. After pet stores, SpringStyles respondents who report Hispanic or Latino ethnicity most often report an unknown location (30.1%; 95% CI: 23.1–37.0%) and non-commercial acquisition for obtaining their turtle (29.5%; 95% CI: 22.5–36.6%). Across all racial and ethnic groups in the SpringStyles survey, a greater proportion of respondents could not recall where they obtained their small turtle compared to Estilos respondents, though the weighted estimates for the Estilos survey and some racial groups in the SpringStyles survey should be interpreted with caution (12).

**Table 3:** Locations of small turtle procurement by race and ethnicity^†,‡^.

% of *n* (95% CI)	Hispanic or Latino		American Indian or Alaska Native	Asian	Black or African American	Native Hawaiian or Pacific Islander	White	Multiple races
	Estilos (*n* = 201)	SpringStyles (*n* = 315)	SpringStyles (*n* = 33)	SpringStyles (*n* = 115)	SpringStyles (*n* = 212)	SpringStyles (*n* = 3)	SpringStyles (*n* = 1,096)	SpringStyles (*n* = 78)
Pet Store	64.3% (54.3–74.4%)	36.4% (29.0–43.9%)	15.9% (0–34.2%)^¶^	28.5% (16.3–40.7%)	23.5% (16.2–30.7%)	0	29.1% (26.2–32.0%)	25.5% (13.7–37.4%)
Transient vendor^§^	19.5% (10.8–28.1%)	11.8% (7.1–16.5%)	1.5% (0–4.5%)^¶^	6.1% (0–12.5%)^¶^	13.3% (7.5–19.2%)	6.0% (0–19.1%)^¶^	2.8% (1.6–4.1%)	3.7% (0.5–7.0%)^¶^
Non-commercial acquisition^§^	21.4% (14.2–28.6%)	29.5% (22.5–36.6%)	37.2% (13.7–60.6%)^¶^	34.3% (21.4–47.2%)	31.2% (23.0–39.3%)	23.9% (0–62.6%)^¶^	38.7% (35.5–41.9%)	54.6% (41.5–67.8%)
Unknown^§^	4.9% (0–10.8%)^¶^	30.1% (23.1–37.0%)	48.6% (23.6–73.6%)^¶^	32.4% (20.4–44.5%)	33.9% (25.5–42.3%)	76.1% (37.4–100%)^¶^	27.9% (24.9–30.9%)	23.8% (12.2–35.3%)

^†^Respondents in this table were limited to those who currently own or previously owned a small turtle, and respondents were allowed to select all purchase locations that apply.

^‡^Percentages and confidence intervals are rounded to the nearest tenth place. Percentages represent the proportion of respondents that selected the specific demographic and turtle-based question answer choice.

^§^Response options were combined to improve estimate stability. The category of transient vendor includes responses from “Roadside Vendor” and “Flea Market.” The category of non-commercial aquisition includes responses from “Caught in the Wild,” “Animal Shelter,” and “Friend or Family Member.” The category of Unknown includes responses from “Other,” “Unknown,” and respondents who have ever owned a small turtle but did not provide an answer of where their small turtle was obtained.

^¶^Relative standard error was greater than 30% of the weighted estimate; therefore, these weighted estimates and 95% CI’s should be interpreted with caution (8, 12).

## Discussion

This descriptive analysis characterizes small turtle ownership across demographic groups in the United States and in relation to several Hispanic cultural characteristics. In the Estilos survey, Hispanic people reported higher small turtle ownership compared with all racial and ethnic groups in the SpringStyles survey. It should be emphasized that both of these surveys are inherently different regarding methodology, and factors including year of administration, recruitment strategies, question verbiage, or other differences between the two surveys could have impacted the comparability between Estilos and SpringStyles. Overall, the frequency of current or previous small turtle ownership among Hispanic people aligns with demographic trends observed in previous multistate small turtle-associated salmonellosis outbreaks, which have consistently disproportionately affected Hispanic people (4, 6, 13).

Neither of the surveys collected data to explain the differences in small turtle ownership observed across racial and ethnic groups. Furthermore, neither survey characterized respondents’ purpose for ownership, whether that was for companionship, animal breeding, animal exhibition, or some other reason. Regardless, educational resources produced by public health agencies must effectively reach groups at highest risk across different languages, communication styles, and information sources. When Hispanic and Latino populations are overrepresented in enteric outbreaks, CDC investigators have disseminated outbreak and prevention materials in Spanish (14, 15). News media platforms that use languages preferred by the specific populations affected could also be leveraged. In the Estilos survey, people who reported ever owning a small turtle most frequently used Spanish media and mostly or only spoke Spanish at home. The findings of this study reinforce the need for multi-pronged, iterative efforts to understand motivations behind small turtle ownership and to communicate about the risk of *Salmonella* infection from small turtles to the general population and those at greatest risk. Additional actionable strategies to achieve these goals could include public health agency collaboration with pet stores to provide human and animal health informational resources in multiple languages, veterinary and human medical providers sharing preventative health information, or social media campaigns distributing information in multiple languages.

The disaggregated analysis of the Estilos’ acculturation model (a metric that calculates the acculturation level of individuals by ranking and summarizing years lived in the United States, cultural identification, language spoke at home, and media habits) highlights the granularity of underlying cultural identities that may contribute to disparate health outcomes (16–19). Among all respondents in the Estilos survey, most identify as “somewhat/much closer” to Hispanic or Latino culture than US culture, as did Estilos respondents who ever owned a small turtle. To effectively investigate or deliver prevention information about enteric disease risks associated with small turtle ownership, it may be useful to incorporate Hispanic and United States cultural influences and use both English and Spanish language when developing prevention materials.

Among respondents who have ever owned small turtles, pet stores were the most common source of small turtles for Hispanic people. Similarly, people who reported Asian, Black or African American, White, or multiple races also obtained their small turtles from pet stores. Federal and some state regulations prohibit sale of small turtles as pets in the United States, but sale of small turtles at brick-and-mortar pet stores has been documented and has contributed to a small turtle-associated salmonellosis outbreak previously (5). The high proportion of survey respondents who reported purchasing small turtles from pet stores suggests that local and state officials could assess the availability of small turtles at pet stores in their jurisdisctions and engage in appropriate regulatory action. Furthermore, across racial and ethnic groups surveyed, respondents tended to report obtaining their small turtles from transient vendors such as roadside or flea market vendors less commonly than obtaining their small turtles from pet stores or through non-commercial means of acquisition. However, transient vendors more frequently have been identified as the source of turtles in small turtle-associated salmonellosis outbreaks than pet store locations (4, 15). Additional research is needed to determine whether turtles sold from transient vendors represent a greater risk to public health compared to turtles obtained from other sources and, if so, what factors contribute to that risk. However, it can be challenging to identify and communicate with these vendors because patients might not remember where they interacted with such vendors or because these vendors might move locations (6, 10, 20).

The findings in this report are subject to several limitations. First, the two surveys analyzed are inherently different with varying administration years, recruitment methodologies, question formulation, available answer choices, and weighting. These differences could have affected the observed frequencies and limit the direct comparability of the results between the two surveys. Second, respondents who ever owned a small turtle include respondents who report previous small turtle ownership. Because it is unknown how long ago respondents owned their turtles, this limits our ability to inform the contemporary state of small turtle ownership and sale in the United States. Furthermore, these turtles may have been obtained and owned in a different country that did not have regulations on small turtles. Similarly, previous ownership in respondents older than 48 years could have occurred before federal or state bans were enacted in the United States. Therefore, the high frequency of people who reported purchasing their turtle at a pet store might not exclusively represent illegal domestic sale of small turtles as pets. Next, ethnicity of SpringStyles respondents who were categorized as multiple race is unknown due to the structure of the survey’s race and ethnicity question. Recommendations may apply to those who are multiple race, but without ethnicity information, we cannot make definitive conclusions about these respondents. Further, language spoken at home (as collected by Estilos) may not fully represent the language variability at the household level and does not distinguish between “preferred” or “primary” language. The lack of information pertaining to language variability in this study should be considered when developing language-based prevention approaches. Next, sample sizes of several subgroups were small (i.e., weighted estimates had >30% relative standard error), and estimates were unstable (8, 12). Similarly, the sample sizes of both surveys and the frequency of those who reported ever owning turtles precluded further stratification of survey results. Future studies that plan to use weighted survey data should consider anticipated exposure frequencies in addition to survey sample size; rare exposures are less likely to be interpretable after survey weighting. Finally, both surveys were distributed to the public for voluntary participation in exchange for cash-equivalent rewards. As such, these do not represent random sampling of the general population and could introduce selection or response biases. These are additionally important to consider given the rarity of small turtle exposure as respondents might differ from the general population.

Prevention efforts for small turtle-associated salmonellosis could focus on the Hispanic and Latino populations that most commonly own small turtles and are identified in outbreaks of *Salmonella* illnesses associated with small turtles. Public health communicators may consider this study’s findings about the cultural characteristics of Hispanic or Latino small turtle owners by including relevant culturally-specific approaches in developing and evaluating communications content. Circulating content in the language used for media consumption through appropriate channels and in the language spoken at home may reach the population most affected by small turtle-associated salmonellosis. While our study did not yield data on other ethno-linguistic groups, we believe that a similar approach could be useful in messaging to other over-represented populations in enteric disease outbreaks. Future studies may consider taking a qualitative approach to investigate why Hispanic people choose small turtles as pets, message testing of existing prevention materials to evaluate tailoring and dissemination strategies, and assessing population-level awareness of turtle-associated salmonellosis and knowledge, attitudes, and practices related to prevention strategies.

## Data Availability

The data analyzed in this study is subject to the following licenses/restrictions: The data that support the findings of this study are subject to licensing from Porter Novelli Public Services, Inc. Restrictions apply to the availability of these data, which were used under license for this study. Requests to access these datasets should be directed to eza@cdc.gov.
