# Supplementary material for: Ethnic differences of small turtle ownership in the United States: a descriptive analysis of Porter Novelli Estilos and SpringStyles surveys, 2021 and 2023
Source: Front Public Health. 2026 Oct 15;14:1920888. doi: 10.3389/fpubh.2026.1920888 (PMC13725822; doi:10.3389/fpubh.2026.1920888)
Supplement: Supplementary file 1 [file Table_1.DOCX]

## **Supplementary Material**

**Supplementary Table S1.** Comparable questions asked in Estilos (2021) and SpringStyles (2023) surveys.

| **Topic** | **Survey** | **Question** | **Answer Choices** |
| --- | --- | --- | --- |
| Small Turtle Ownership | Estilos | Please select all of the following statements that apply to your pet turtle ownership. | a. I currently have a small turtle, less than the size of an egg b. I previously had a small turtle, less than the size of an egg c. My current/previous small turtle lives/lived indoors d. I have never owned a small turtle |
|  | SpringStyles | Have you ever had either a small turtle (less than the size of an egg) or a large turtle (greater than the size of an egg) in your household? | a. Currently have b. Previously had c. Never had d. Refused |
| Small Turtle Purchase Location | Estilos | Where was/were your small turtle(s) purchased or obtained from? | a. Pet store b. Road-side vendor c. Flea market d. Caught in the wild e. Animal shelter or animal rescue f. Obtained from friend or family member g. Purchase location other/unknown |
|  | SpringStyles | If someone in your household currently or previously had a small turtle, where did you get it? | a. Never had a small turtle in my house b. Pet store c. Roadside vendor d. Flea market e. Caught in the wild f. Animal shelter or animal rescue g. Friend or family member h. Other i. Unknown j. Refused |
| Race and Ethnicity | SpringStyles | What race or ethnicity do you most identify with? | a. American Indian or Alaska Native b. Asian c. Black or African American d. Native Hawaiian or Pacific Islander e. Hispanic or Latino f. White g. I identify equally with more than one |
| Education Level | Estilos |  | a. Less than high school b. High school c. Some college d. Bachelor degree or higher |
|  | SpringStyles |  | a. Less than HS b. HS c. Some College d. Bachelor or higher |
| Parental Status | Estilos | Do you currently have children under the age of 18?  Starting with your youngest child, please list the ages of your 5 youngest children. | a. Yes b. No   [Free response] |
|  | SpringStyles | Do you currently have children under the age of 18?  Starting with your youngest child, please list the ages of your 3 youngest children. | a. Yes b. No   [Free response] |
| Cultural Identification | Estilos | Thinking of your overall cultural identification, would you say you feel …? | a. Much closer to Hispanic/Latino Culture b. Somewhat closer to Hispanic/Latino Culture c. Equally close to both cultures d. Somewhat closer to U.S. Culture e. Much closer to U.S. Culture |
| Years Living in the United States | Estilos | How many years have you lived in the United States? | a. 0-4 b. 5-9 c. 10-14 d. 15-19 e. 20 years or more |
| Media Habits | Estilos | Thinking of your media habits, including television, radio, newspapers and magazines or the internet, would you say you use… | a. Spanish media only b. Spanish media mostly c. Spanish and English media equally d. English media mostly e. English media only |
| Language | Estilos | What language do you usually speak at home? | a. Spanish only b. Spanish mostly c. Spanish and English equally d. English mostly e. English only |
